# Curcumin and piperine emulsomes as combinational treatment approach to enhance the anti-biofilm, anti-microbial, and wound healing activities of curcumin

**DOI:** 10.1007/s13197-025-06327-7

**Published:** 2025-05-30

**Authors:** Nithya Ramalingam, Subhashini Swaminathan, Raghavendra Ramalingam

**Affiliations:** 1 Department of Biotechnology, SRM Institute of Science and Technology, Kattankulathur, Chengalpattu, Tamil Nadu 603 203 India; 2 Department of Prosthodontics and Implantology, Saveetha Dental College and Hospital, Saveetha Institute of Medical and Technical Sciences (SIMATS), Chennai, 600077 India

**Keywords:** Curcumin-Piperine, Emulsomes, Drug delivery, Bioavailability, Antibiofilm, Wound healing

## Abstract

Curcumin, a polyphenolic substance from turmeric, is known for its anti-inflammatory, antioxidant, and antimicrobial properties. However, its poor bioavailability limits clinical application. Piperine, an alkaloid from black pepper, enhances curcumin’s bioavailability. Advanced drug delivery systems like emulsomes can improve curcumin’s efficacy by providing targeted and prolonged delivery. This study investigated the combined effects of curcumin and piperine emulsomes on anti-biofilm, antimicrobial, and wound healing activities. The physicochemical properties of the emulsomes were characterized using dynamic light scattering (DLS), transmission electron microscopy (TEM), and X-ray diffraction (XRD). The antibacterial activity against biofilm-forming pathogens, including *Staphylococcus aureus* and *Pseudomonas aeruginosa*, was evaluated using the well diffusion assay. Kinetics and mechanisms of antibacterial action were analyzed with time-kill assays and membrane degradation studies. Anti-biofilm activity was assessed using the crystal violet assay, and wound healing efficacy was tested in an in vitro scratch assay with 3T3 fibroblast cells. In all assays, the curcumin and piperine emulsomes demonstrated superior activity compared to curcumin alone and blank controls. Hence, we can conclude that curcumin and piperine emulsomes can significantly enhance curcumin’s antimicrobial, anti-biofilm, and wound healing effects, offering a promising combinational treatment approach for microbial infections and wound management.

**Supplementary Information:**

The online version contains supplementary material available at https://doi.org/10.1007/s13197-025-06327-7.

## Highlights

- Enhanced Curcumin Bioavailability: Piperine significantly boosts curcumin’s absorption, overcoming poor bioavailability.
- Novel Emulsomes Formulation: Curcumin-piperine emulsomes exhibit stable, targeted drug delivery.
- Anti-biofilm Efficacy: The combination of curcumin + piperine emulsomes inhibits biofilm formation by *Staphylococcus aureus* and *Pseudomonas aeruginosa*.
- Superior Wound Healing: Curcumin + Piperine emulsomes enhance cell migration, proliferation, and collagen synthesis.
- Sustained Antimicrobial Activity: Curcumin + Piperine emulsomes provide prolonged antimicrobial effects.

**Supplementary Information:**

The online version contains supplementary material available at https://doi.org/10.1007/s13197-025-06327-7.

## Introduction

The emergence of antibiotic-resistant bacterial infections has become a significant public health challenge, necessitating the development of novel therapeutic strategies. Among the various approaches being explored, the use of natural compounds with potent antibacterial properties has gained considerable attention. This research focuses on the combined application of curcumin and piperine encapsulated within emulsomes to evaluate their antimicrobial and antibiofilm activity against two notorious pathogens: *Staphylococcus aureus* and *Pseudomonas aeruginosa*, along with the study of wound healing activity of the synthesized emulsomes.

Curcumin is a vibrant yellow polyphenolic compound derived from the rhizome of *Curcuma longa* (turmeric) (Lestari and Indrayanto 2014). It has been extensively researched for its remarkable therapeutic properties including antibacterial, anti-inflammatory, antioxidant, and anticancer activities. Traditional medicine systems, particularly in Asian countries, have utilized curcumin for centuries due to its medicinal benefits (Kocaadam and Şanlier 2017). Despite its extensive therapeutic potential, curcumin’s clinical application is significantly hampered by its poor bioavailability, resulting from rapid metabolism and low absorption rate in the gastrointestinal tract (Dei Cas and Ghidoni 2019). To address this challenge, researchers have explored various adjuncts that can enhance curcumin’s bioavailability. Piperine, an alkaloid extracted from black pepper (*Piper nigrum*), has emerged as a promising adjunct due to its ability to inhibit certain metabolic enzymes and increase the absorption of various substances in the gut (Tiwari et al. 2020). Curcumin undergoes rapid liver and intestinal wall metabolism, primarily through glucuronidation. Piperine inhibits UDP-glucuronosyltransferase (UGT) enzymes responsible for this metabolic pathway, thereby slowing down curcumin’s metabolism and increasing its concentration in the bloodstream (Althobaiti et al. 2022). Piperine also inhibits P-glycoprotein, a transporter protein that pumps foreign substances out of cells, preventing the efflux of curcumin from intestinal cells back into the gut lumen, and enhancing its absorption (Tripathi et al. 2022).

Studies have shown that the combination of curcumin with piperine can substantially improve the bioavailability of curcumin, thereby enhancing its therapeutic efficacy (Yüksel et al. 2023, Pratti et al. 2024). Both compounds possess anti-inflammatory and antioxidant properties, and their combination shows promise in enhancing curcumin’s anti-cancer effects (Martins et al. 2024; Sahar et al. 2024). Piperine also stimulates digestive enzymes, improving digestion and nutrient absorption, including curcumin. Together, they can protect against gastrointestinal disorders, reduce inflammation, and promote gut healing (Chen et al. 2020). It also provides neuroprotective benefits by crossing the blood-brain barrier more effectively, aiding in the protection against neurodegenerative diseases and cognitive decline (Kundu et al. 2016).

Enhanced drug delivery systems are a requirement because of the disadvantages of conventional drug delivery methods which include limitations such as poor bioavailability, systemic toxicity, and non-specific targeting (Senapati et al. 2018). The modernized drug delivery system offers various advantages such as improved drug stability, increased bioavailability, and targeted delivery to specific tissues or cells. Techniques like nano-delivery systems use nanosized materials for precise and controlled delivery of therapeutic agents. They also make it easier to transport medications to previously unreachable locations, opening new avenues for treating several types of illnesses (Patra et al. 2018).

The development of such delivery systems is particularly important in treating infections caused by biofilm-forming pathogens, where high and sustained concentrations of the therapeutic agent are necessary to disrupt and eradicate biofilms effectively (Kumar et al. 2023).

Among the various advanced drug delivery systems, emulsomes have gained considerable attention for their ability to increase the bioavailability and therapeutic efficacy of hydrophobic compounds like curcumin. Emulsomes are lipid-based vesicular systems that combine the properties of both liposomes and emulsions (Singh et al. 2023). They consist of an inner solid lipid core surrounded by phospholipid bilayers, providing a stable and biocompatible matrix for encapsulating hydrophobic drugs. Emulsomes offer several benefits as drug delivery systems. Their lipid-based composition enhances the solubility and stability of encapsulated drugs, while the vesicular structure facilitates controlled and sustained release (Afreen 2020). Additionally, emulsomes can be engineered to target specific tissues or cells, improving the selectivity and efficacy of the therapeutic agent. Emulsomes can protect these compounds from metabolic degradation, enhance their absorption, and ensure prolonged circulation in the bloodstream, making them a promising platform for delivering curcumin in the treatment of various diseases (Kumar et al. 2022).

Biofilms are organized bacterial colonies that adhere to surfaces by embedding themselves in an extracellular polymeric substance (EPS) matrix. The formation of biofilms involves initial microbial adhesion, microcolony formation, maturation, and dispersion (Rather et al. 2021). In medical settings, biofilms are highly significant because they frequently form on medical devices such as catheters, prosthetic joints, heart valves, and human tissues. These biofilm-associated infections are notoriously difficult to treat due to their high resistance to antibiotics and immune system evasion (Talsma 2007). The EPS matrix protects the microbes, while the proximity of cells facilitates the exchange of resistance genes, leading to chronic conditions and increased morbidity and mortality (2021). *Staphylococcus aureus* and *Pseudomonas aeruginosa* are prominent pathogens in healthcare-associated infections (HAIs) (Sievert et al. 2013). *S. aureus* is a Gram-positive coccus found in clusters and is part of the skin and nasal flora, causing infections from minor skin issues to severe conditions like sepsis and pneumonia. *P. aeruginosa* is a Gram-negative rod found in water and soil, thriving in moist environments like hospitals. Both bacteria are known for forming biofilms, which complicate treatment by enhancing resistance to antibiotics and the host immune response.

In healthcare settings, *S. aureus* and *P. aeruginosa* produce major problems in device-related infections. Biofilms act as barriers to antibiotic penetration, harbor persistent cells, and facilitate the spread of resistance genes. Consequently, biofilm-associated infections by these pathogens are persistent and difficult to treat, requiring prolonged and aggressive treatment strategies, including combination therapies, high antibiotic doses, and sometimes surgical removal of infected devices (Tran et al. 2023). Treating biofilm-forming strains of *S. aureus* and *P. aeruginosa* is challenging due to their robust defenses. Biofilms inhibit antibiotic penetration and promote resistance gene exchange, leading to multidrug-resistant strains. *S. aureus* biofilms cause chronic infections on medical devices and tissues, often needing surgical intervention. *P. aeruginosa* biofilms are problematic in cystic fibrosis, chronic wounds, and burns. Both bacteria evade the immune system through metabolic alterations and quorum sensing. The resilience of these biofilms demands innovative therapies and effective antibiofilm agents.

Wound healing research has seen significant advancements with the exploration of novel therapeutic agents, one of the most promising being the combination of curcumin and piperine in emulsomes formulations. Curcumin is renowned for its anti-inflammatory, antimicrobial, and antioxidant properties, crucial in accelerating wound healing. Piperine, enhances the bioavailability of curcumin, thus amplifying its therapeutic effects. In cell line studies, applying curcumin and piperine emulsomes has significantly enhanced wound healing parameters. These formulations promote cell migration, proliferation, and collagen synthesis, essential processes for effective wound closure and tissue regeneration (Kumari et al. 2022). The synergistic effect of curcumin and piperine also mitigates inflammation at the wound site, reduces oxidative stress, and inhibits bacterial growth, creating an optimal environment for healing (Abu-Hijleh et al. 2024). Additionally, studies have shown that emulsomes can protect the active ingredients from degradation, thereby sustaining their therapeutic concentrations at the wound site over extended periods (Patil et al. 2023). Moreover, the use of curcumin has been linked to improved angiogenesis, and the formation of new blood vessels, which is vital for delivering nutrients and oxygen to the healing tissue. This aspect of wound healing is crucial for the regeneration of functional tissue and the prevention of chronic wounds (Wang and Chen 2019). The potential of these emulsomes in promoting wound healing not only holds promise for better clinical outcomes but also reduces the reliance on conventional treatments that often have limitations due to antibiotic resistance and side effects. Therefore, the ongoing research and development of curcumin and piperine emulsomes could pave the way for more effective, safer, and faster wound healing therapies, potentially revolutionizing wound care practices.

## Materials and methods

### Materials

Curcumin, Piperine, glyceryl tripalmitate (tripalmitin, purity ≥ 99%), Cholesterol (≥%99), Chloroform (≥ 99.8%), Polysorbate 80 (T80), Mueller Hinton agar, Trypticase soy broth, Crystal violet dye, Phosphate buffer saline, Brain Heart Infusion agar, Congo Red powder, Sucrose, Sodium acetate, Acetic acid were procured from SRL Pvt. Ltd., India. 3T3 (fibroblast cell line) was obtained from the National Centre for Cell Sciences (NCCS), Pune, India. Cells were maintained in the logarithmic phase of growth in Dulbecco’s modified eagle medium (DMEM) supplemented with 10% (v/v) heat inactivated fetal bovine serum (FBS), 100 U/mL penicillin, 100 µg/mL streptomycin. They were maintained at 37 °C with 5% CO_2_ in 95% air humidified incubator. *Staphylococcus aureus* and *Pseudomonas aeruginosa* clinical strains were provided by the Department of Microbiology, SRM Medical College, India.

### Emulsomes Preparation

**Fig. 1:**
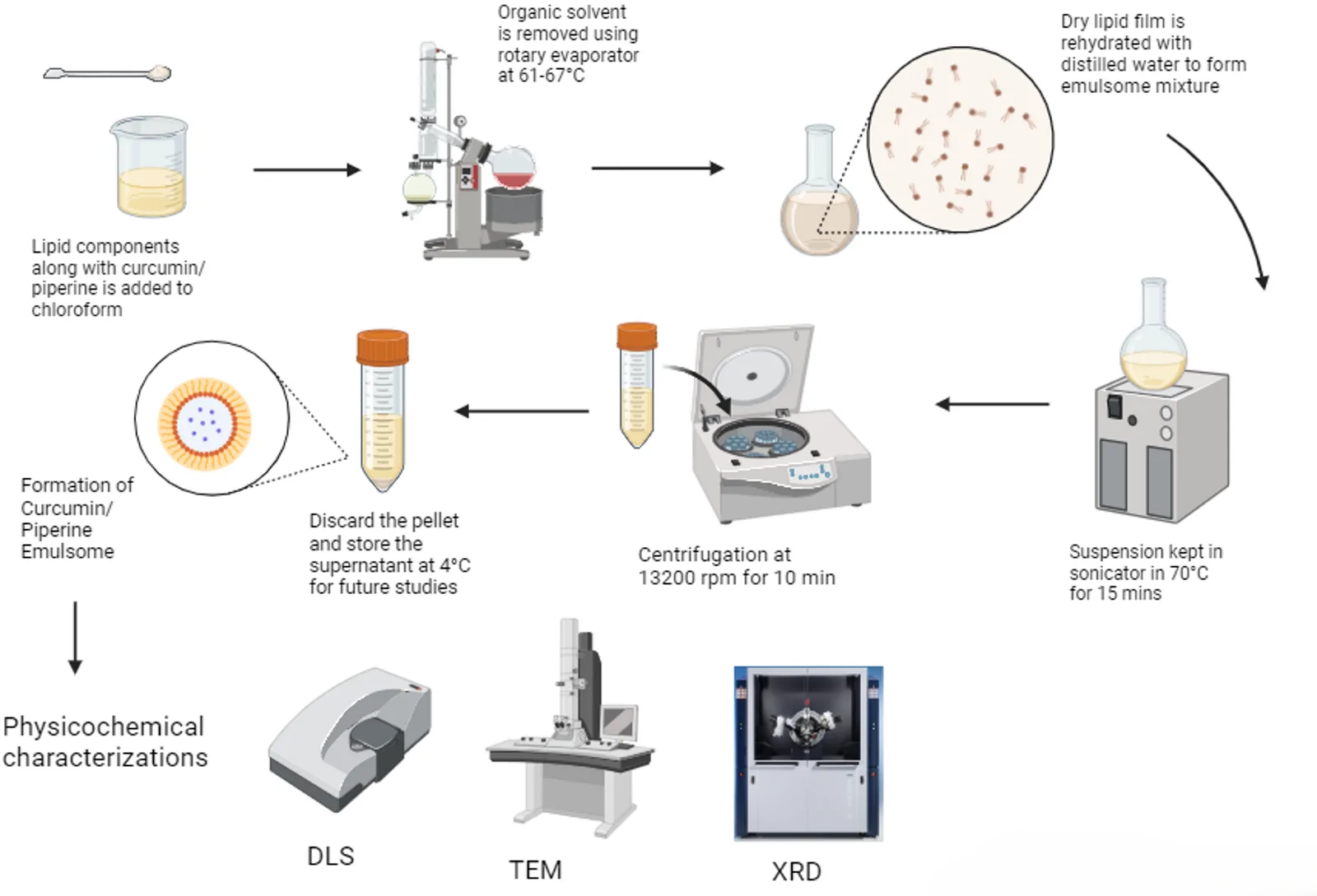
Synthesis of Curcumin and Piperine Emulsomes and its characterizations

Preparation of the emulsomes has been done by applying the method described by (Bolat et al. 2020) (Fig. 1). For curcumin emulsomes, lipids including 20 mg of tripalmitin, 0.6 mg of cholesterol, and 20 mg of curcumin (2wt%) were mixed in 2 mL of chloroform (organic solvent). For curcumin + piperine emulsomes, lipids including 20 mg of tripalmitin, 0.6 mg of cholesterol, and 10 mg of curcumin (1 wt%) and 10 mg of piperine (1 wt%) were mixed in 2 mL of chloroform (organic solvent). The solution was stirred with an emulsifying agent (Tween 80) to create an emulsion. Then, the mixture was placed in a rotary evaporator at 60–65 °C to remove the organic solvent, resulting in a dry lipid film. The dry lipid film was rehydrated with 5 mL of distilled water by gentle mixing or swirling. Next, the lipid suspension was placed in an ultrasonication bath heated to 70 °C for 15 min until homogenized. After homogenization, the suspension was centrifuged at 16,100 x g for 10 min to separate the pellet and supernatant. Discard the pellet and store the supernatant in the fridge at 4 °C until further use. Blank Emulsomes were prepared following the same procedure without adding curcumin or piperine.

### Characterization of emulsomes using TEM

TEM offers valuable insights into their size, shape, and internal structure at the nanoscale level. A small droplet of the emulsomes suspension was placed onto a TEM grid. Excess liquid was carefully removed to create a thin film of the emulsomes suspension on the grid. The grid was dried in a hot air oven to ensure the emulsomes were immobilized in their native state. By comparing TEM images of blank, curcumin, and piperine emulsomes, differences in their physical properties can be assessed, and gain insights into how the encapsulation of curcumin and piperine influences the overall structure of the emulsomes.

### Characterization of emulsomes using DLS

DLS provides valuable insights into their physical properties, such as particle size, stability, and polydispersity. Emulsomes samples are dispersed in a suitable solvent or medium, followed by the measurement of scattered light intensity as a function of time. For blank emulsomes, DLS can elucidate the inherent characteristics of the lipid-based carriers without any encapsulated payload. When applied to curcumin and piperine-loaded emulsomes, DLS helps assess the effect of payload incorporation on particle size and stability.

### Characterization of emulsomes using XRD

XRD serves as a valuable tool for elucidating their molecular arrangements and identifying any crystalline phases that are present. First, the sample is prepared by finely grinding the material into a powder to ensure uniformity and maximize surface area for accurate measurement. The powdered sample is then loaded onto a sample holder, ensuring a flat and smooth surface. By comparing the diffraction patterns of the individual components (curcumin powder, piperine powder) to those of the physical mixture and emulsomes, XRD assesses any changes in crystalline structure induced by formulation processes such as mixing and encapsulation. Differences in peak intensities, positions, and shapes can indicate alterations in the molecular arrangement, phase transitions, or the presence of new crystalline phases arising from interactions between the components or with the carrier matrix.

### Tube assay

Tube assay is a qualitative method used for the detection of biofilm as described by (Harika et al. 2020). 20 mL of Trypticase soy broth was prepared and 10 mL of broth was poured in each test tube and inoculated with the chosen bacterial strains. The tubes were incubated for 24 h at 37 °C. After incubation, the contents were discarded, and the tubes were rinsed with PBS and allowed to air-dry. The tubes were stained with 0.1% crystal violet dye for 10 min. After being cleaned with distilled water, the stained tubes were inverted and allowed to air dry. After air drying, the tubes are observed for visible staining of the dye in walls, ring interface, and bottom, indicating biofilm production.

### Congo red assay

The Congo red agar method followed by (Kali et al. 2016) was used for the detection of biofilm in bacteria isolates. 100 mL BHI agar was prepared and autoclaved. 80 mg of Congo red powder and 5% sucrose solution were added to this, creating the Congo Red BHI agar solution. Then the Congo Red BHI agar solution was poured into Petri plates and let solidify. After that, the plates were inoculated with the bacterial strains and were incubated aerobically for 48 h at 37 °C. After 2 days of incubation, the Petri plates were observed. Bacterial strains that produced black colonies with crystalline texture were considered biofilm producers and hence selected for detection of the antibiofilm action of curcumin and piperine.

### Microtiter plate assay using crystal Violet dye

To assess the effectiveness of the emulsomes samples in preventing biofilm formation, (Harika et al. 2020) method of Microtiter plate assay was done in 96 well plates using crystal violet in a modified way. The concentration range of the emulsomes used for the study was 0 to 100 µg/mL. Each well was filled with 100 µL of sterile Trypticase soy broth medium in rows B, C, E, and F from wells 1–11. Wells 1–2 received 100 µL of emulsomes: curcumin emulsomes (2 wt%) in rows B & C, and curcumin (1 wt%) + piperine emulsomes (1 wt%) (8:2 v/v) in rows E & F Column 2 was mixed, and 100 µL was transferred sequentially up to well 10. Subsequently, 20 µL of bacterial culture (OD 0.5) was added to wells 1–11. It was followed by 80 µL of fresh medium to the wells. Well 12 served as a sterility control with only 200 µL of sterile medium, and well 11 was a positive control with only TSB and bacteria. Plates were incubated for 24 h at 37 °C. After incubation, the well contents were discarded and washed with 0.2 mL of PBS. The adhering sessile bacteria were fixated with 2% sodium acetate and 0.1% (w/v) crystal violet was used to stain the bacteria. After using deionized water to remove extra stains, the plates were allowed to air dry. 33% acetic acid is used to wash the wells. With a microtiter plate reader, optical density was determined at 630 nm.

Using the following formula, the percentage of biofilm inhibition was determined:

$$\:\%\:Biofilm\:Inhibition\:=\frac{Control\:OD-Test\:OD}{Control\:OD}\:\times\:100$$

### Well diffusion assay

The antimicrobial activity of curcumin and piperine emulsomes was determined using the well diffusion assay. 100 mL Mueller Hinton agar was prepared. Once the agar was solidified, 100 µL of the bacterial strain was spread evenly across the agar plate surface. Wells were created in the agar plates using a sterile tool. The wells were carefully filled with the test samples (30 µL each), consisting of the positive control (antibiotics like ciprofloxacin and aztreonam), negative control (blank emulsomes), free curcumin, curcumin emulsomes (2 wt%), and curcumin (1 wt%) + piperine (1 wt%) emulsomes. The concentration of ciprofloxacin, aztreonam, curcumin and piperine was 5 µg, 30 µg, 2 wt%, and 1 wt% respectively. The plates were incubated for 18–24 h at 37 °C. Inhibition zones around the wells were measured after the incubation period.

### Time kill kinetics assay

Time Kill assay was performed to evaluate the bactericidal/bacteriostatic activity of the curcumin and piperine emulsomes over time, and thereby helps to determine its effectiveness in reducing bacterial populations. The bacterial suspensions were first diluted with MHB broth and incubated for 12 h at 37 °C to ensure proper growth. Following incubation, 100 µL of the bacterial strain was transferred to each well in a 96-well plate. Each well contained 100 µL of blank or curcumin and piperine emulsomes at a 50% (w/w) concentration. For control wells, we used bacterial suspensions without any treatment to compare the effects of the emulsomes. Using a plate reader, the absorbance of every well was measured at 600 nm five separate times: 0,2,4,6, and 8 h. This allowed for the assessment of bacterial growth and the potential inhibitory effects of the curcumin and piperine emulsomes over time when compared to blank emulsomes.

### Membrane degradation assay

The membrane degradation assay was performed to evaluate the action and effectiveness of curcumin and piperine emulsomes on the bacterial cell membrane integrity. To analyze the release of cellular material, both *Staphylococcus aureus* and *Pseudomonas aeruginosa* were utilized. Bacterial suspension was prepared in PBS. For the experimental setup, 1 mL of this bacterial suspension was treated with 50% curcumin + piperine emulsomes concentration (80:20 ratio). As a control, an equivalent volume of blank emulsomes was used to treat the bacterial cells. The treatment was applied for various time intervals, specifically 0, 10, 20, 30, 60, and 90 min. Post-treatment, the bacterial cells were centrifuged at 1207 x g for 2 min. Following centrifugation, the supernatant was carefully collected and its absorbance was measured at 260 nm.

### In-vitro scratch wound assay

To determine the wound healing properties, a scratch wound assay was performed in vitro. This experiment was performed according to the previously reported and standardized protocol by (Liang et al. 2007). 3T3 cell were seeded in 6-well plates (1 × 10^5^ cells/well) and grown until reached a confluence of 90–95%, in the optimum culture conditions. After reaching the confluency, a scratch was made midway through the monolayer of cells using a P10 pipette tip to resemble a wound, and fresh medium was used to clean the wells with any cell debris. Negative control cells did not receive any treatment. The wounded area was exposed to curcumin and piperine emulsomes samples for 24–48 h at 37 °C in the incubator, ensuring an atmosphere of 5% CO_2_. The closure of the wound scratch was analyzed using a digital inverted microscope at different periods by capturing images at time 0 h, 24 h, and 48 h (static imaging) to monitor the healing process. Using the ImageJ processing program, the difference between the wound widths at T0 and T1/T2 was measured to quantify the closure of the scratch. The scratch closure rate was measured as described by (Felice et al. 2015), using the formula below:

$$\:\rm{SCR}\:=\:\left(\right(T0\:\--\:T1/T2)\:/\:T0)\:\times\:\:100$$

where T0 is the scratch area at time 0 h and T1/T2 is the scratch area at 24 and 48 h.

## Results & discussion

### Transmission electron microscope (TEM)

**Fig. 2:**
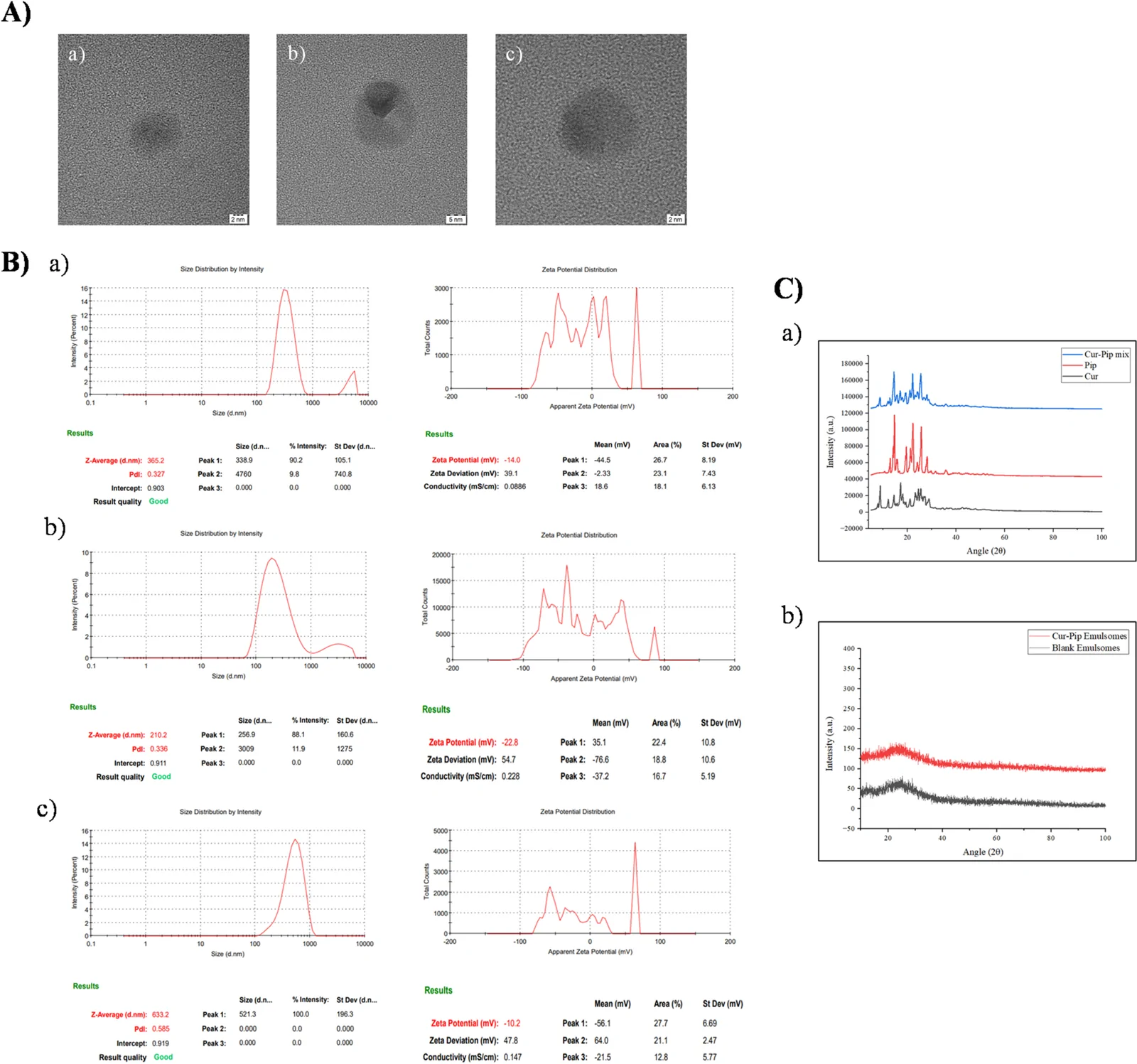
**A)** TEM images of a) blank emulsomes (b) curcumin emulsomes and (c) curcumin + piperine emulsomes **B)** Particle size distribution & zeta potential of (a) blank (b) curcumin and (c) curcumin + piperine emulsomes **C)** XRD analysis of (a) raw curcumin, piperine & its physical mixture (b) blank & curcumin + piperine emulsomes

TEM images of various emulsomes are depicted in Fig. 2A. The obtained images reveal that the emulsomes were in the nanometer size range and exhibit a spherical shape with a distinct core-shell structure, making them suitable for drug delivery due to enhanced cellular uptake and bioavailability. The images indicate minimal aggregation, with the particles mostly separate and well-dispersed. Emulsomes loaded with curcumin and piperine show dark regions within the core, indicating successful encapsulation of the drugs. This is due to the higher electron density of the dense core compared to the surrounding lipid shell, resulting in greater absorption of electrons and a darker image for the core. In contrast, blank emulsomes lack these dark cores, confirming the absence of encapsulated drugs.

### Dynamic light scattering (DLS)

DLS results (Fig. 2B) reveal that curcumin has the smallest particle size (210.2 nm) and moderate zeta potential (-22.8 mV), with a relatively uniform size distribution (PDI = 0.336). Piperine has the largest particle size (633.2 nm) and low zeta potential (-10.2 mV), with the highest PDI (0.585), indicating a heterogeneous size distribution. Blank has moderate particle size (365.2 nm) and the moderate zeta potential (-14.0 mV), with a relatively uniform size distribution (PDI = 0.327). Overall, the Blank formulation shows the best potential for stability, while the curcumin formulation has the advantage of smaller particle size. The Piperine formulation has a larger size and broader distribution.

### X-ray diffraction (XRD)

XRD spectrum of curcumin, piperine, and emulsomes are depicted in Fig. 2C. The results confirm that raw curcumin and piperine are in crystalline form, and their physical mixture retains the crystallinity of both components. No significant interactions occurred between curcumin and piperine when mixed physically. All samples show clear, sharp peaks with minimal noise due to their high crystalline nature. For all three samples, the peaks at 2θ = 10–30° are prominent. Curcumin has a low-intensity peak compared to Piperine indicating piperine is more crystalline. For curcumin-piperine emulsomes and the blank formulation, the XRD analysis confirms that they are predominantly amorphous, due to the absence of sharp peaks. The broad peaks around 2θ = 20° in both samples indicate the lack of crystalline material. Curcumin-piperine emulsomes have a more amorphous nature, likely due to the encapsulation of curcumin and piperine, which will destabilize the lipid structure of emulsomes. The amorphous state is often desirable in drug formulations because it can enhance solubility and bioavailability.

### Tube assay

**Fig. 3:**
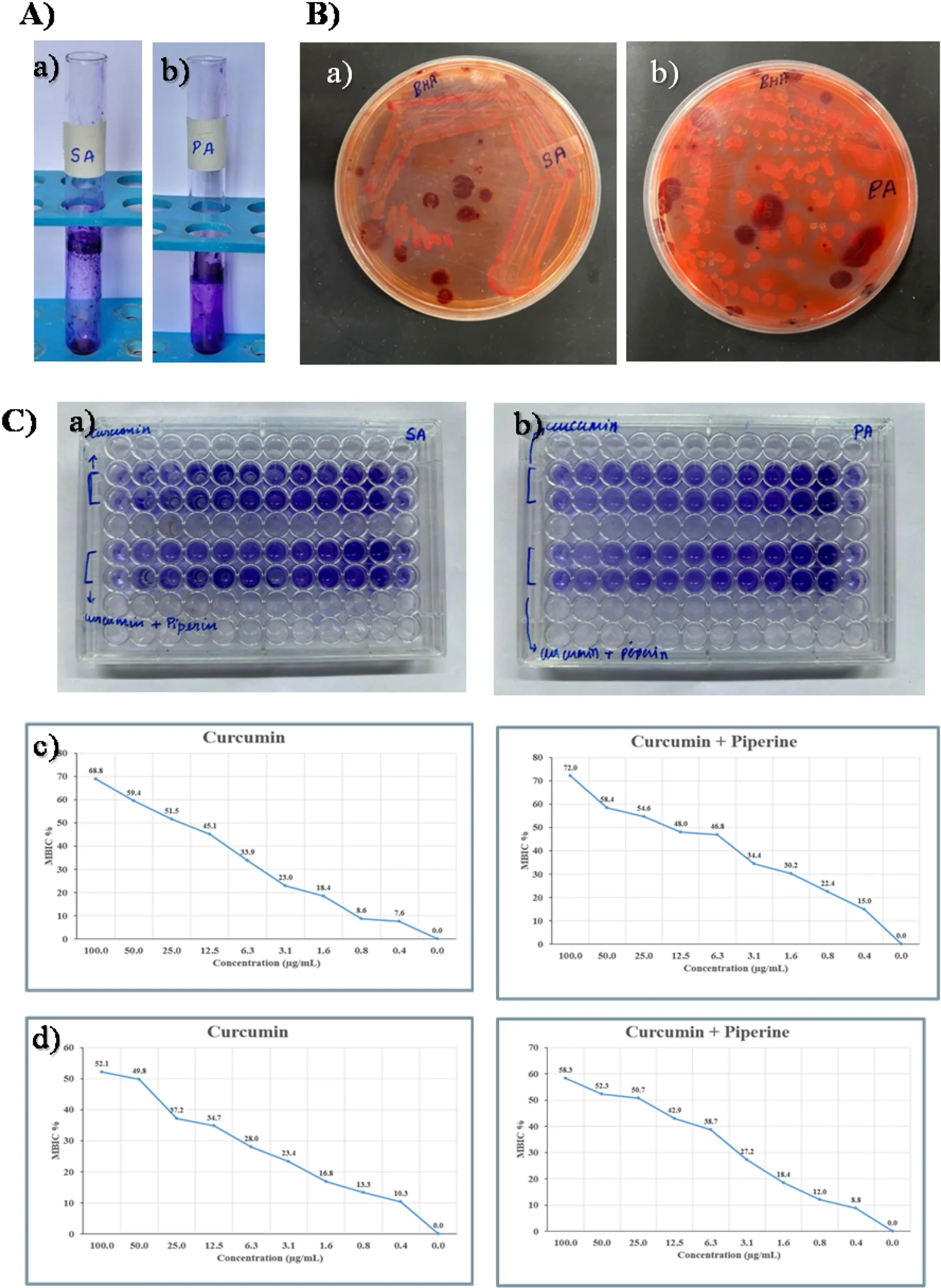
**A)** Tube Assay showing biofilms of (a) *S. aureus* (b) *P. aeruginosa ***B)** Congo Red Assay showing biofilms of (a) *S. aureus* (b) *P. aeruginosa***C)** Microtiter plate assay showing anti-biofilm activity of (a) *S. aureus* (b) *P. aeruginosa* (c) Biofilm Inhibition rate in *S. aureus* (d) Biofilm Inhibition rate in *P. aeruginosa*

Rings were formed at the interference of the liquid medium and visible layers were formed on walls and the bottom of the tube in both the bacterial strains (Fig. 3A). Hence both are considered to be biofilm-producers. Non-biofilm producers do not have any indications in the test tubes for biofilm.

### Congo red assay

Congo Red dye binds to the extracellular polysaccharides in biofilm and produces dark red/black colonies with crystalline texture, indicating biofilm production. Pink-colored colonies indicate the absence of biofilm production. Since dark red colonies are seen in both bacteria, they are considered to be biofilm producers (Fig. 3B).

### Microtiter plate assay using crystal Violet dye

Darker wells indicate high biofilm formation and lighter wells indicate less biofilm formation due to the action of curcumin and piperine emulsomes (Fig. 3C). The crystal violet dye binds to the biofilm matrix and cells, to quantify the biofilm production visually. The emulsomes show a significant reduction of biofilm production in both bacteria with increasing concentration. For both bacteria, curcumin emulsomes alone start with a slightly lower MBIC% (68.8% and 52.1%) at 100 µg/mL compared to curcumin + piperine emulsomes (72% and 58.3%). The biofilm inhibition (%) against the *S. aureus* and *P. aeruginosa* at various concentrations of emulsomes is shown in Tables 1 and 2, respectively. The presence of piperine seems to enhance the antibiofilm activity slightly as indicated by higher initial MBIC%. The trend provided by the graph was consistent with the tabular data. Hence it clearly shows that anti-biofilm activity will be dose-dependent with higher concentrations of emulsomes showing greater efficiency in inhibiting biofilm formation.

**Table 1:** Biofilm Inhibition rate in *S. aureus*

Sl. No.	Concentration (µg/mL)	Biofilm Inhibition %	
		Curcumin (2 wt%)	Curcumin (1 wt %) + Piperine (1 wt%)
1.	100	68.8	72.0
2.	50	59.4	58.4
3.	25	51.5	54.6
4.	12.5	45.1	48.0
5.	6.3	33.9	46.8
6.	3.1	23.0	34.4
7.	1.6	18.4	30.2
8.	0.8	8.6	22.4
9.	0.4	7.6	15.0
10.	0	0.0	0.0

**Table 2:** Biofilm Inhibition rate in *P. aeruginosa*

Sl. No.	Concentration (µg/mL)	Biofilm Inhibition %	
		Curcumin (2 wt%)	Curcumin (1 wt %) + Piperine (1 wt%)
1.	100	52.1	58.3
2.	50	49.8	52.3
3.	25	37.2	50.7
4.	12.5	34.7	42.9
5.	6.3	28.0	38.7
6.	3.1	23.4	27.2
7.	1.6	16.8	18.4
8.	0.8	13.3	12.0
9.	0.4	10.3	8.8
10.	0	0.0	0.0

### Well diffusion assay

**Fig. 4:**
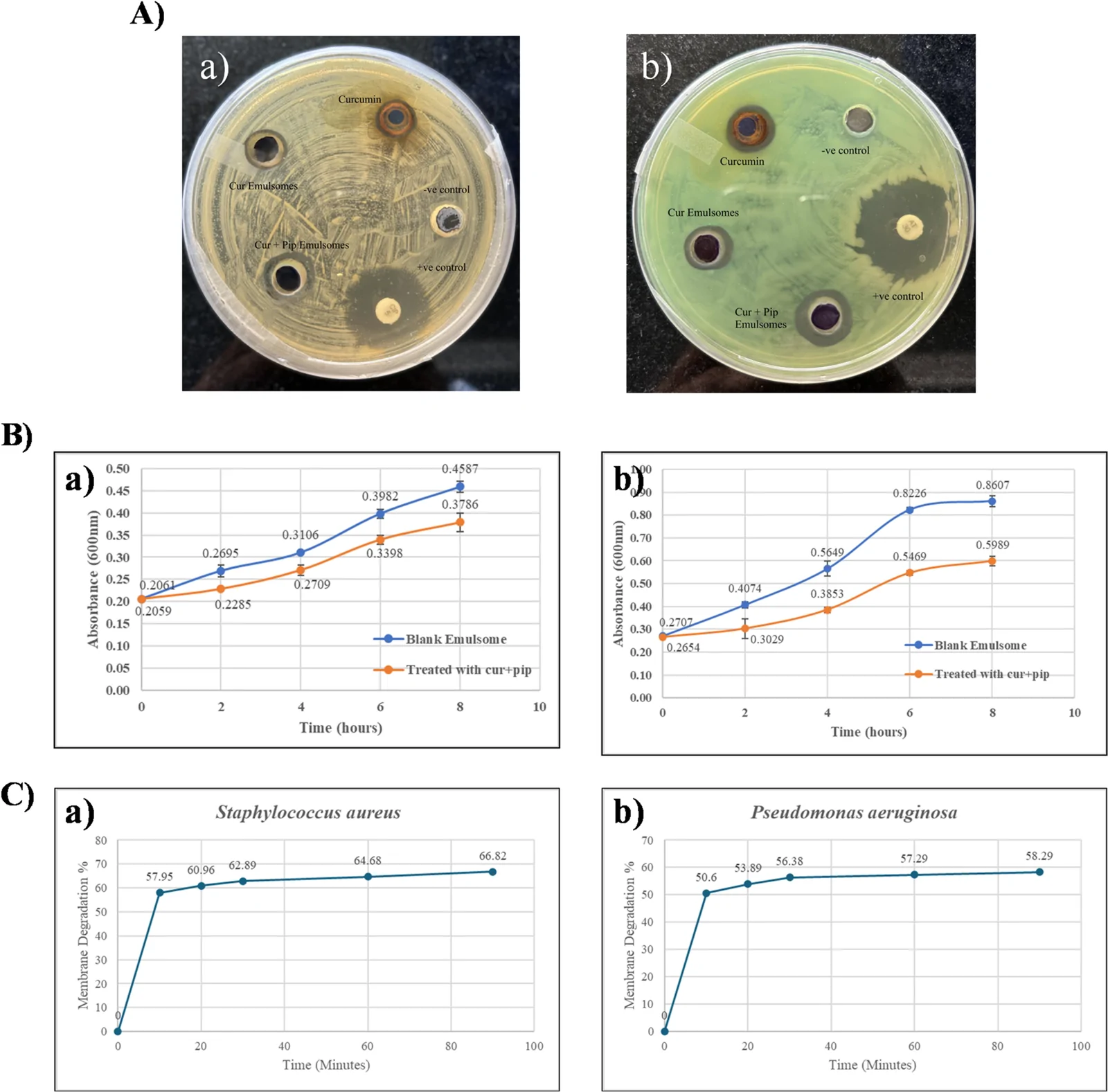
**A)** Well Diffusion assay showing anti-microbial activity of (a) *S. aureus* (b) *P. aeruginosa***B)** Time Kill Kinetics rate in (a) *S. aureus* (b) *P. aeruginosa***C)** Membrane Degradation rate in (a) *S. aureus* (b) *P. aeruginosa*

Both the well diffusion plates show huge inhibition zones at the positive control by antibiotic application (Ciprofloxacin for *S. aureus* (23 mm) and Aztreonam for *P. aeruginosa* (30 mm)) but do not show any inhibition zones in the negative control well (blank emulsomes) due to lack of antimicrobial agent (Fig. 4A). Both the bacteria showed inhibition zones in all sample wells. When compared between curcumin, curcumin emulsomes, and curcumin + piperine emulsomes, the curcumin piperine emulsomes combination showed the highest zone of inhibition, thereby indicating that piperine provides an additional antibacterial effect to curcumin (Table S4).

### Time kill kinetics assay

Results show that in both the bacteria, it shows a trend where there was an increase of microbial growth occurring over time in the case of both blank and curcumin + piperine emulsomes (Fig. 4B). While both the control and treated ones show bacterial growth, the main key observation was the difference in growth dynamics between these two emulsomes. The treatment with curcumin + piperine was partially effective as it slows down the growth of bacteria when compared to the blank control but does not completely inhibit its growth. This assay was done to find whether the emulsomes have a bactericidal/bacteriostatic nature against the bacteria. In this case, the emulsomes are not bactericidal but partially bacteriostatic, where they inhibit the bacterial growth slowly over time instead of completely killing them.

### Membrane degradation assay

The percentage of membrane degradation increases with respect to time, which implies that the curcumin + piperine progressively damages the bacterial membrane (Fig. 4C). Higher Optical Density represents a higher rate of membrane degradation. Since membrane integrity is crucial for bacterial survival, the increasing membrane degradation correlates with bacterial killing. In the graph, both bacteria show a huge difference in OD between the 0th minute and 10th minute, indicating that most of the membrane degradation rate occurs between those time periods.

### In-vitro scratch wound assay

**Fig. 5:**
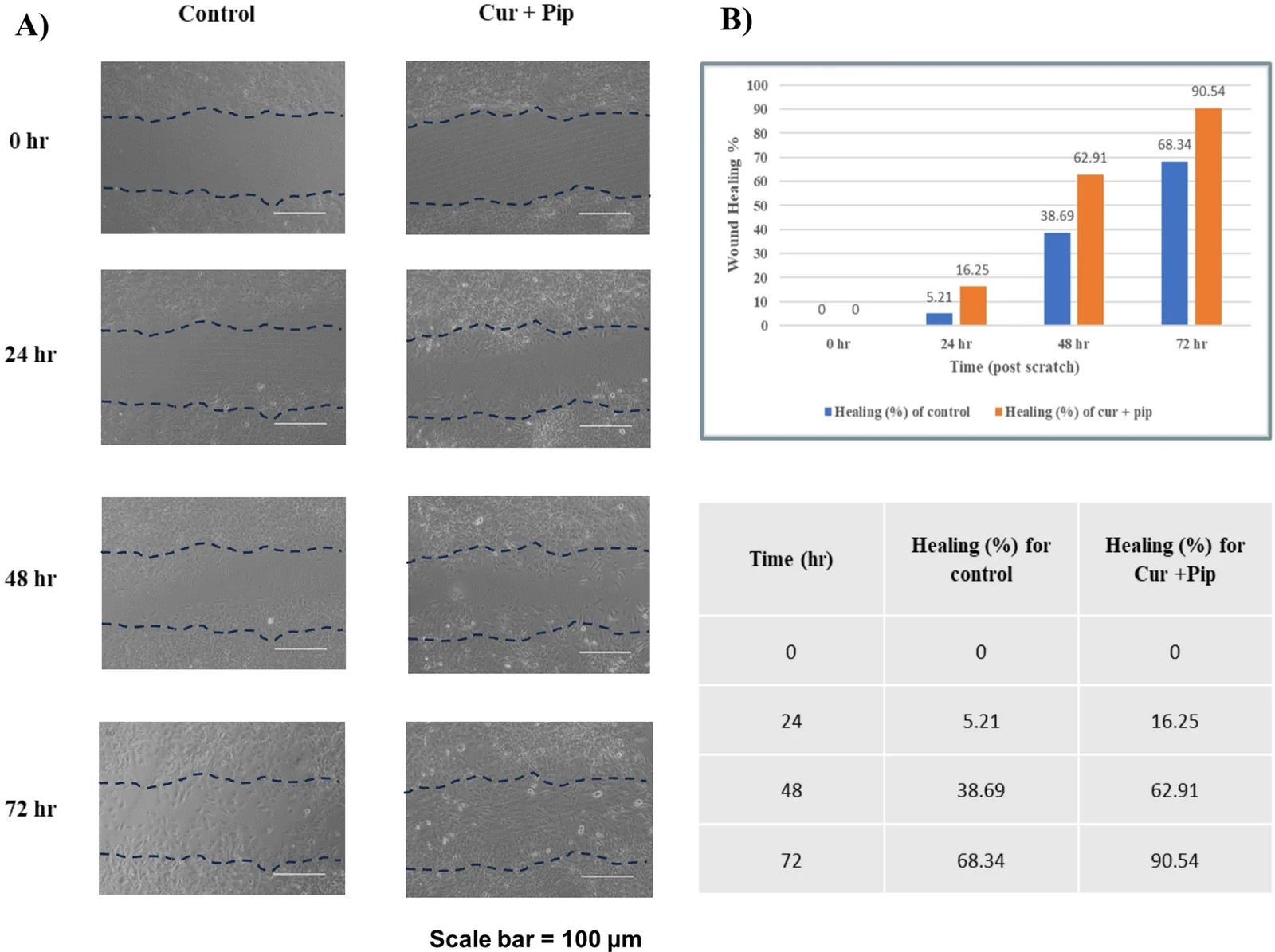
**(A)** In-vitro Scratch wound assay for control and curcumin + piperine emulsomes treated 3T3 cells at 0, 24, 48 and 72th hour **(B)** Wound Healing rate for control and curcumin + piperine emulsomes

Results are observed in the digital images captured by an Inverted microscope. In Fig. 5A, at the 0th hr, the initial wound was visible with a wide gap between the two cell fronts. After 24 h, a slight closure of the wound gap was visible, indicating the beginning of the healing process. When compared to the control group, the treated group shows a more noticeable reduction in the wound gap, suggesting a faster healing process. After 48 h, the wound gap has reduced further than before. In the treated group, the cells migrated more towards the centre when compared to the control group at the same time point. At 72nd hr, the wound in control was nearly closed, indicating substantial cell migration and proliferation. The wound in the treated group appears completely closed, potentially more so than the control group, indicating enhanced wound healing due to the curcumin and piperine emulsomes treatment. Wound treated with curcumin + piperine emulsomes shows a high wound healing percentage at each time point when compared to the control group (Fig. 5B). Also, curcumin + piperine treatment accelerates and enhances the wound healing process, due to the combinational antimicrobial, anti-inflammatory, and tissue regenerative properties of curcumin and piperine.

## Conclusion

The research conducted provides significant advancements in the formulation and application of curcumin and piperine emulsomes aimed at enhancing their bioavailability, stability, and therapeutic efficacy. The study primarily focused on the combinational anti-biofilm and antimicrobial effects of these emulsomes against two major pathogens, *Staphylococcus aureus*, and *Pseudomonas aeruginosa*, as well as their potential to enhance wound healing properties. Physicochemical characterizations confirmed the structure and stability of the synthesized emulsomes, while extensive evaluations demonstrated their efficacy. The results showed significant anti-biofilm and anti-microbial activities, with the curcumin-piperine emulsomes inhibiting biofilm formation and promoting bacterial cell membrane degradation. The wound scratch assay revealed enhanced wound healing properties, evidenced by improved cell migration and proliferation. The key takeaway is the promising potential of curcumin-piperine emulsomes for treating biofilm-associated infections and enhancing wound healing. Future research should focus on optimizing the formulation, exploring synergistic effects with conventional antibiotics, and evaluating the safety profiles of these emulsomes. Incorporating them into medical devices or topical formulations could significantly reduce healthcare-associated infections and improve patient outcomes. Developing targeted delivery systems to localize treatment to infected or wounded areas could reduce side effects and increase therapeutic effectiveness. Ultimately, clinical trials will be necessary to validate their efficacy and safety in humans, paving the way for regulatory approvals and commercialization. Integrating curcumin and piperine emulsomes with wound care products could revolutionize the management of chronic wounds and other difficult-to-treat conditions, with potential applications extending to veterinary medicine.

## Electronic supplementary material

Below is the link to the electronic supplementary material.

Supplementary Material 1

## Data Availability

Data will be made available on reasonable request.

## Abbreviations

DLS: Dynamic Light SCATTERING
XRD: X-Ray Diffraction
FBS: Fetal Bovine Serum
EPS: Extracellular Polymeric Substances
PDI: Polydispersity Index
MHB: Mueller Hinton Broth
TSB: Trypticase Soy Broth
BHI: Brain Heart Infusion Broth
PBS: Phosphate-buffered saline
UGT: UDP-glucuronosyltransferase
COVID-19: Coronavirus disease 2019
DMEM: Dulbecco’s Modified Eagle Medium
