# Supplementary material for: Curcumin and piperine emulsomes as combinational treatment approach to enhance the anti-biofilm, anti-microbial, and wound healing activities of curcumin
Source: J Food Sci Technol. 2025 May 30;63(12):2299–312. doi: 10.1007/s13197-025-06327-7 (PMC13751900; doi:10.1007/s13197-025-06327-7)
Supplement: Supplementary file 1 [file 13197_2025_6327_MOESM1_ESM.docx]

**Curcumin and Piperine Emulsomes as Combinational Treatment Approach to Enhance the Anti-biofilm, Anti-microbial, and Wound Healing Activities of Curcumin**

**Supplementary Tables:**

**Table S1:** Time Kill Kinetics rate in *S. aureus*

| Time  (hour) | Blank Emulsomes  (O.D) | Treated with cur +pip  (O.D) |
| --- | --- | --- |
| 0 | 0.2061 | 0.2059 |
| 2 | 0.2695 | 0.2285 |
| 4 | 0.3106 | 0.2709 |
| 6 | 0.3982 | 0.3398 |
| 8 | 0.4587 | 0.3786 |

**Table S2:** Time Kill Kinetics rate in *P. aeruginosa*

| Time  (hour) | Blank Emulsomes  (O.D) | Treated with cur + pip  (O.D) |
| --- | --- | --- |
| 0 | 0.2707 | 0.2654 |
| 2 | 0.4074 | 0.3029 |
| 4 | 0.5649 | 0.3853 |
| 6 | 0.8226 | 0.5469 |
| 8 | 0.8607 | 0.5989 |

**Table S3:** Membrane Degradation rate in *S. aureus* and *P. aeruginosa*

| S. No | Time (Min) | Membrane Degradation %  (*S. aureus*) | Membrane Degradation %  (*P. aeruginosa*) |
| --- | --- | --- | --- |
| 1. | 0 | 17.98 | 18.63 |
| 2. | 10 | 57.95 | 50.60 |
| 3. | 20 | 60.96 | 53.89 |
| 4. | 30 | 62.89 | 56.38 |
| 5. | 60 | 64.68 | 57.29 |
| 6. | 90 | 66.82 | 58.29 |

**Table S4:** Zone of Inhibition for Various Antimicrobial Treatments Against *S. aureus* and *P. aeruginosa*

| **DESCRIPTION** | **Zone of Inhibition** |
| --- | --- |
| Positive control (ciprofloxacin) in *S. aureus* | Diameter: 23 mm |
| Positive control (Aztreonam) in *P. aeruginosa* | Diameter: 30 mm |
| Curcumin in *S. aureus* | Diameter: 11 mm |
| Curcumin in *P. aeruginosa* | Diameter: 13 mm |
| Curcumin Emulsomes in *S. aureus* | Diameter: 12 mm |
| Curcumin Emulsomes in *P. aeruginosa* | Diameter: 14 mm |
| Curcumin + Piperine Emulsomes in *S. aureus* | Diameter: 15 mm |
| Curcumin + Piperine Emulsomes in *P. aeruginosa* | Diameter: 16 mm |
